# Impact of bone marrow-derived mesenchymal stem cells on remodeling the lung injury induced by lipopolysaccharides in mice

**DOI:** 10.4155/fsoa-2016-0036

**Published:** 2017-01-17

**Authors:** Mouchira M Mohi El-Din, Laila A Rashed, Mohi A Mahmoud Haridy, Atef Mohamed Khalil, Mohamed A Mohamed Albadry

**Affiliations:** 1 Pathology & Clinical Pathology Department, Faculty of Veterinary Medicine, South Valley University, Qena, Egypt; 2 Biochemistry & Molecular Biology Department, Medicine Faculty, Cairo University, Cairo, Egypt

**Keywords:** cytokines, flow cytometry, fluorescent technique, histopathology, immunohistochemistry, lipolysaccharide, lungs, mesenchymal stem cells, mice

## Abstract

**Aim:**

This study evaluated the potential of bone marrow derived mesenchymal stem cells (MSCs) to regulate cytokines and remodel the lung induced by lipopolysaccharide (LPS; O-antigen).

**Materials & methods:**

A group of mice (n = 21) was inoculated intraperitoneally with one dose 0.1 ml containing 0.025 mg LPS/mouse, and another treated intravenously with one dose of labeling bone marrow derived MSCs at 7.5 × 10^5^ cell/mouse 4 h after LPS injection. All animals were sacrificed on the 1st, 7th and 14th days post-injection.

**Results:**

MSCs increased the level of IL-10 with suppression of TNF-α, decrease of collagen fibers and renewal of alveolar type I cells, together with lung tissue remodeling.

**Conclusion:**

MSCs were shown to modulate inflammatory cytokines (TNF-α and IL-10) and to differentiate into alveolar type I cells, which prevented fibrosis in lung tissue from LPS-treated mice.

## Lay abstract

This study sought to confirm the remodeling effect of mesenchymal stem cells (MSCs) on lungs injury resulting from lipopolysaccharide infection, as lipopolysaccharide has an important role in acute lung injury pathogenesis. MSCs decreased the level of TNF-α and increased the level of antinflammatory cells (IL-10), leading to prevention of fibrosis with renewal of alveolar type I cells. This research aids our understanding of the utility of MSCs in chronic lung injury treatment.

**Figure 1.**
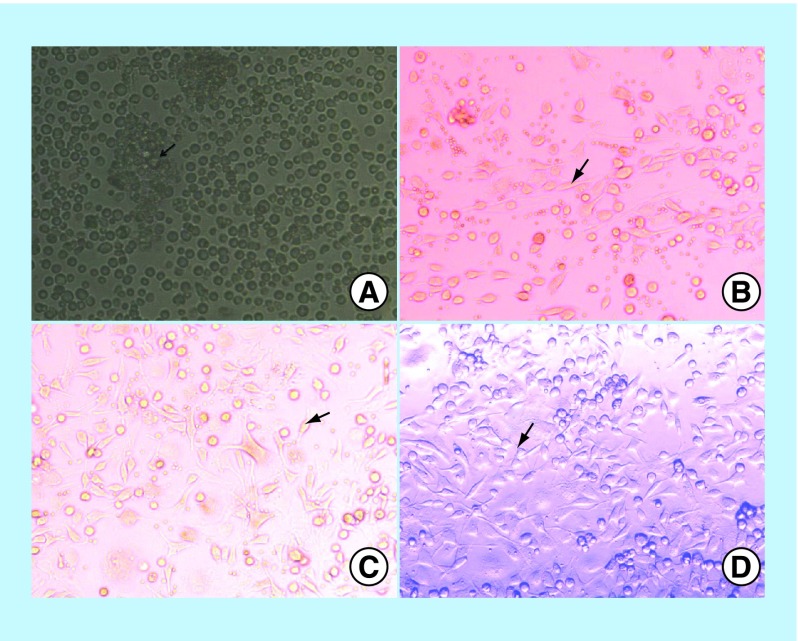
**Bone marrow–mesenchymal stem cells.** Patches of aggregated cells (arrow) before incubation **(A)**. Elongated, fusiform, and spindle cells at the 3rd day post injection (DPI), (arrow) **(B)**. Relatively homogenous cell cultures ofmesenchymal stem cells resemble the fibroblast morphology on the 7th DPI **(C)**. Relatively homogenous cell cultures on the 14th DPI (x200).

**Figure 2.**
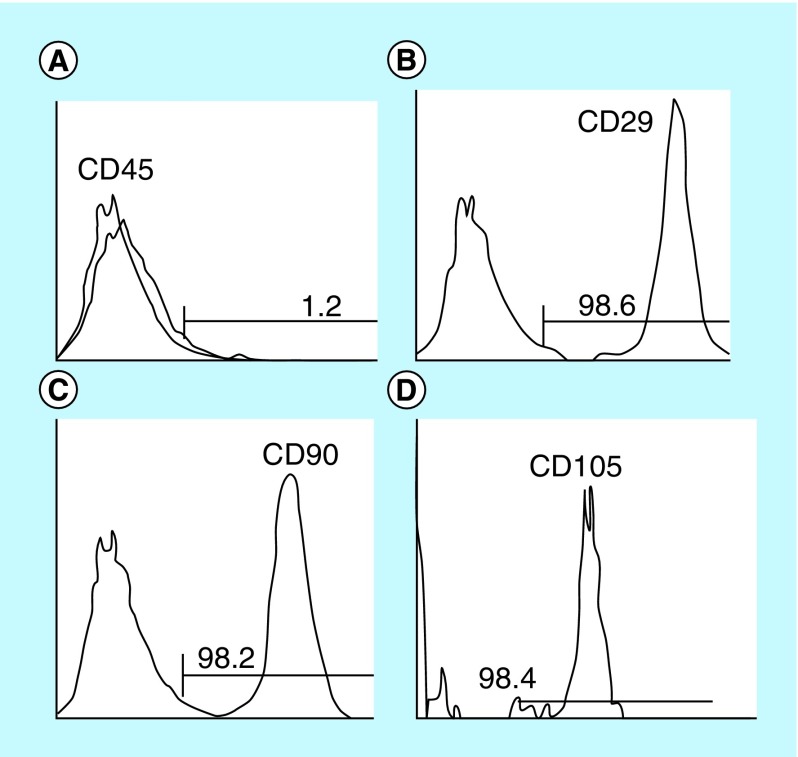
**Flow cytometric characterization analysis of bone marrow-mesenchymal stem cells.** The cells were uniformly negative for CD45 **(A)** and positive for CD29 **(B)**, CD90 **(C)**, and CD105 antibodies **(D)**.

**Figure 3.**
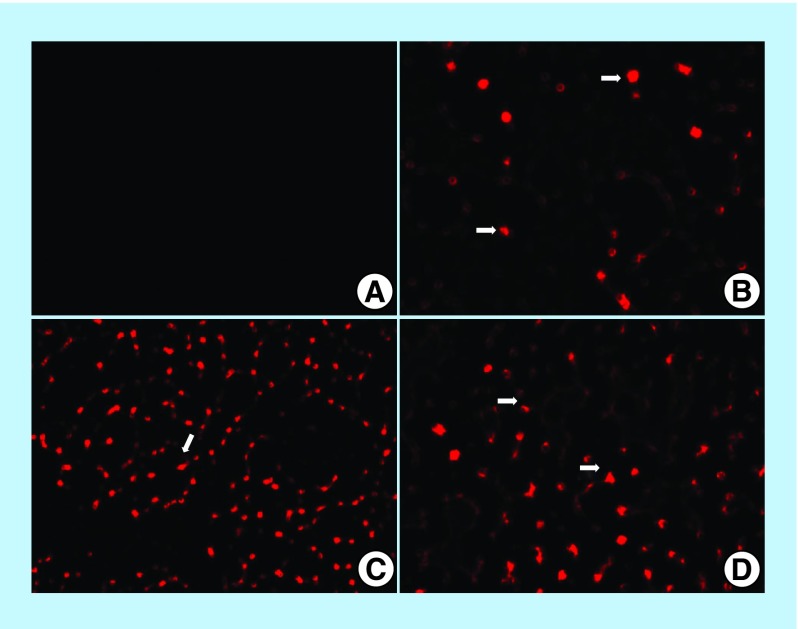
**Photomicrograph of fluorescent microscopy for the lung sections of mice.** No auto-fluorescence was observed in the unstained (gp 1) and no treated group (gp 2) **(A)**. In the treated group (gp 3), with PKH26 labeled-MSCs on the 1st day post injection (DPI), red fluorescent spots (arrows) **(B)**. Diffuse of red fluorescent spots (arrow) on the 7th DPI **(C)**. Variable sized red fluorescent spots show homing of the cells in the lung tissue (arrows) on the 14th DPI (x200) **(D)**.

**Figure 4.**
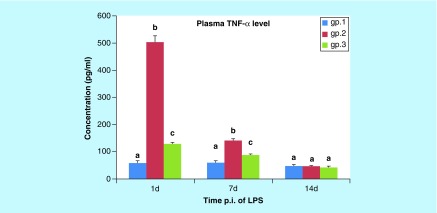
**Histogram of the mean values (± standard error of the mean) of the TNF-a level in the mice plasma (gps 1, 2, and 3) on the 1st, 7th, and 14th days post injection.** Different letters indicate a significant change when p < 0.05. LPS: Lipopolysaccharide; p.i: Post-inoculation.

**Figure 5.**
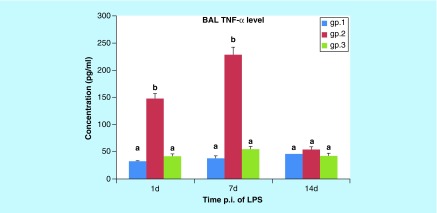
**Histogram of the mean values (± standard error of the mean) of the TNF-a level in the BAL of mice (gps 1, 2, and 3) on the 1st, 7th, and 14th days post injection.** Different letters indicates significant change when (p < 0.05). BAL: Bronchoalveolar lavage; LPS: Lipopolysaccharide; p.i: Post-inoculation.

**Figure 6.**
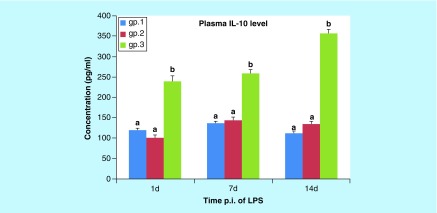
**Histogram of the mean values (± standard error of the mean) of the IL-10 level in the plasma of mice (gps 1, 2, and 3) on the 1st, 7th, and 14th days post injection.** Different letters indicate significant change when p < 0.05. LPS: Lipopolysaccharide; p.i: Post-inoculation.

**Figure 7.**
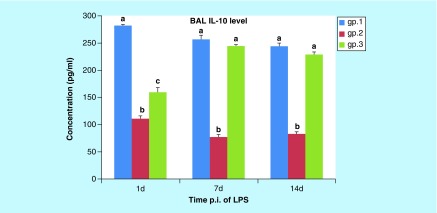
**Histogram of the mean values (± standard error of the mean) of the IL-10 level in BAL of mice (gps 1, 2, and 3) on the 1st, 7th, and 14th days post injection.** Different letters indicate significant change when p < 0.05. BAL: Bronchoalveolar lavage; LPS: Lipopolysaccharide; p.i: Post-inoculation.

**Figure 8.**
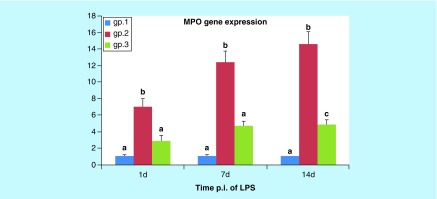
**Histogram of the mean values of *MPO* gene expression in the lungs of mice (gps 1, 2, and 3) on the 1st, 7th, and 14th days post injection.** Different letters indicate significant change when p < 0.05. LPS: Lipopolysaccharide; p.i: Post-inoculation.

**Figure 9.**
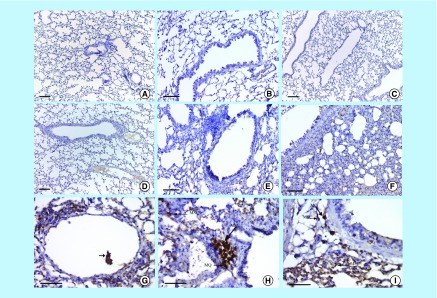
**Lung tissue stained by immunohistochemistry.** No reaction (brown granules) in the lung tissues (gp 1) **(A, C & D)** (bars = 200 µm), with no positive reaction for peroxidase activity in tissues from lipopolysaccharide-treated mice (gp 2) **(B, E & F)** (bars = 100 µm). Mesenchymal stem cells were confirmed from a positive granular brown reaction (arrow) inside the lumen of the alveolar blood vessels, formed as spindle-shape cells (mesenchymal stem cells), and replaced the alveolar epithelial cells and large brown cells (activated macrophages with positive granular brown reaction in their cytoplasm; MQ), which covered the peribronchiolar tissues on the 1st, 7th, and 14th days post injection **(G, H & I)** (All bars = 50 µm).

**Figure 10.**
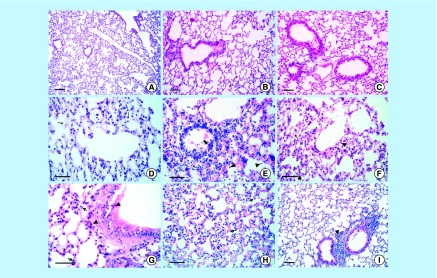
**Lung tissue stained with hematoxylin and eosin.** Normal lung structure (gp 1) **(A, B & C)** (bars = 200 µm). Lipopolysaccharide-induced edematous lungs manifested by vesicle densities in type I epithelial cells of the alveolar septa (gp 2) on the 1st and 14th days post injection (DPI) **(D & F)**. In addition, interstitial pneumonia (arrowhead), fibrosis (thin arrow), and vasculitis (thick arrow) was seen on the 7th DPI (E) (bars = 100 µm). Mesenchymal stem cells restored alveolar epithelial type I cells to their normal structure, with a mild inflammatory reaction noticed in gp 3 on the 1st and 7th DPI **(G & H)** (bars = 50 µm), on the 14th DPI (I) (bar = 200 µm).

**Figure 11.**
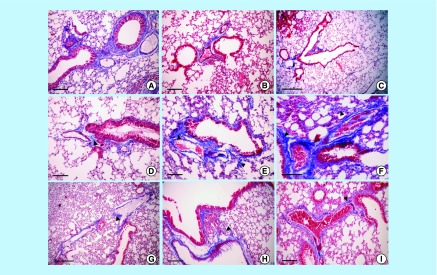
**Lung tissue stained by Masson's trichrome.** Normal structure with no collagen fiber seen in mouse lung tissue (gp 1) **(A & B)** (bars = 100 µm), **(C)** (bar = 200 µm). Lipopolysaccharide-induced fibrosis around the peribronchiolar and perivascular tissues (arrowhead) in gp 2 on the 1st and 7th days post injection **(D & E)** (bars = 100 µm), increased on the 14th day post injection **(F)** (bars = 50 µm). Mesenchymal stem cells limit lipopolysaccharide-induced fibrosis in mouse lung tissue (arrowhead; gp 3) **(G)** (bar = 200 µm), **(H & I)** (bars = 100 µm).

Lipopolysaccharides (LPSs) from Gram-negative bacteria are one of the most potent innate immune-activating stimuli. LPS-inducible genes can regulate the production of cytokines by human monocytes and macrophages [1]. However, LPS also plays an important role in the pathogenesis of acute lung injury in humans and animals [2]. The inflammation induced by acute lung injury causes a disruption of the lung endothelial and epithelial barriers and remains a significant source of morbidity and mortality [3]. Stem cells may be defined as cells that are clonogenic, self-renewing and capable of differentiating into multiple cell lineages [4,5]. Recent findings suggest that exogenous stem cells derived from embryonic and adult tissues can be used for the repair and regeneration of injured or diseased organs, including the lungs [6]. Bone marrow (BM) stem cells can be mobilized to migrate to the injured organ to maintain physiologic hemostasis [7]. These stem cells are derived from adult BM and are able to differentiate into a wide variety of nonhematopoietic cells; they also produce a number of growth factors (cytokines) that are important for tissue repair and remodeling [8,9]. Cell-based therapies using adult stem cells have emerged as a treatment for certain lung diseases, such as emphysema, pulmonary fibrosis, pulmonary hypertension and acute respiratory distress syndrome [10].

The aim of this work was to determine the modulatory effects of BM-derived mesenchymal stem cells (BM-MSCs) on inflammatory reactions and their ability to remodel the lungs, making BM-MSCs a potential therapy for lung injury. The effects of BM-MSCs were detected using ELISA, real-time PCR, immunohistochemistry and histopathological techniques.

## Materials & methods

### Experimental animals

Eighty-four adult male BALB/c albino mice (6 weeks old in age) weighing 20–25 g were purchased from the Animal House of Misr University Sciences and Technology and maintained in a specific pathogen-free environment. The study protocol was approved by the Animal Ethics Committee at South Valley University, Qena, Egypt. All animals were acclimatized in plastic cages (seven animals per cage) inside a well-ventilated room for 1 week prior to the experiment. The animals were maintained under standard conditions (23 ± 3°C temperature, 60–70% relative humidity and a 12 h light/dark cycle), fed a diet of standard commercial pellets and given water *ad libitum*.

### LPSs (O-antigen) from *Escherichia coli*

The LPS product (serotype O127:B8, product number L 3880, stored at 2–8°C, Sigma-Aldrich, MO, USA) was extracted from *E. coli* (source strain ATCC 12740). This LPS serotype has been used to study septic shock [11], to induce NOS activation in murine macrophages [12] and to induce PAF synthesis in rat glomerular mesangial cells [13]. LPSs are made up of a hydrophobic lipid (lipid A, which is responsible for the toxic properties of the molecule), a hydrophilic core polysaccharide chain and a hydrophilic O-antigen polysaccharide side chain.

#### Isolation & characterization of BM-MSCs

Twenty-one adult male albino mice (6 weeks old) underwent BM harvesting by flushing the tibia and femur with Dulbecco's modified Eagle medium (GIBCO/BRL, ThermoFisher Scientific, Paisley, UK). The nucleated cells were isolated with a density gradient (Ficoll/Paque [Amersham Bioscience, NJ, USA]) and then resuspended in complete culture medium supplemented with 1% penicillin-streptomycin and 10% fetal bovine serum (GIBCO/BRL). The cells were incubated in 50-cm culture flask (Falcon, Ciro, Egypt) at 37°C in a 5% humidified CO_2_ incubator for 12–14 days as the primary culture or upon formation of large colonies (80–90% confluent), trypsinized at day 14 with 0.25% trypsin in 1 ml methylenediaminetetraacetic acid (GIBCO/BRL) for 5 min at 37°C [14] and then counted with a hemocytometer. BM-derived MSCs were characterized by their adhesiveness and fusiform shape and identified by staining with surface markers CD29, CD90 and CD105 for MSCs and CD45 for hematopoietic cells using flow cytometry [15].

### Labeling of stem cells with PKH26 dye

MSCs were harvested when the number of suitable viable cells (7.5 × 10^5^)/mice had been obtained and were labeled according to Sigma protocol using a PKH26 red fluorescent kit (Sigma-Aldrich, MO, USA) [16,17]. The cells were centrifuged and washed twice in serum-free media then pelleted and suspended in a dye solution and injected intravenously into a tail vein of the mice. The lung tissue was examined on the 1st, 7th and 14th days post injection to detect and trace the cells using fluorescence microscopy.

#### Experimental design

Sixty-three adult male albino mice were divided into three groups (gp; n = 21).

Group 1 (control gp): The mice were injected intraperitoneally with one 0.1 ml/mouse dose of phosphate buffer saline (PBS);
Group 2 (LPS-infected group): The mice were injected intraperitoneally with one dose of LPS (serotype 0127:B8) at 0.1 ml PBS containing 0.025 mg LPS/mouse) [18,19];
Group 3 (MSC-treated, LPS-infected group): The mice received an intravenous injection of one dose of labeled BM-derived MSCs at a dose of 7.5 ×10^5^ cell/mouse dissolved in PBS 4 h postinoculation along with LPS at a dose of 0.025 mg/mouse).

The animals were examined daily, and clinical signs and mortality and morbidity rates were recorded. The mice were euthanized with xylazine (40 mg/kg) and ketamine (400 mg/kg) [20], blood samples were collected from the medial eye canthus and bronchoalveolar lavages (BALs) were collected from the lungs of sacrificed mice by intratracheal injection with 1 ml/mouse of normal saline. The supernatants of the BAL plasma were used for the estimated TNF-α and IL-10 cytokines evaluation carried out by ELISA.

Two samples from the lungs were collected from all sacrificed animals at 1st, 7th and 14th days post injection (DPI): one sample was used for histopathological and imunohistochemistry analysis and the other was kept frozen at -20°C to determine myeloperoxidase expression by real-time PCR (RT-PCR).

#### Estimation of TNF-α & IL-10 levels in plasma & BAL by ELISA

The quantitative evaluation of the TNF-α and IL-10 levels in the plasma and BAL were carried out using mouse ELISA kits that were purchased from Boster Biological Technology Co. (CA, USA). The test samples and cytokine standards were added to 96-well plates coated with coating antibody, and the plates were then incubated at 37°C for 90 min. After incubation at 37°C for 30 min, the plates were developed with tetramethyl benzidine at 37°C for 20–25 min. The reaction was stopped by the addition of 100 μl of stop solution. The absorbance was measured using an ELISA reader at 450 nm. The concentrations of TNF-α and IL-10 were calculated according to the standard curve using each of the recombinant cytokines in the ELISA kits [21].

#### Detection of myeloperoxidase gene expression by RT-PCR

The RNA was extracted from the lung tissue homogenate using EZ-10 Spin Column Blood Mini-Preps Kit (Bio Basic Inc., ON, Canada). The extracted RNA was then reverse transcribed into complementary DNA (cDNA) using a Reverse Aid First Strand cDNA Synthesis Kit (Thermo Scientific, Vilnius, Lithuania). cDNA was generated from 10 μl of the total RNA that has been extracted with 0.5 μl of Oligo (dt)_18_ primer and 0.5 μl of moloney murine leukemia virus reverse transcriptase enzyme (50 U/μl) for 60 min at 42°C in programmed thermal cycler (HYBAID, MA, USA). The relative abundance of mRNA species was assessed with an ABI prism 7500-sequence detector system (Applied Biosystems, CA, USA). PCR primers were designed using Gene Runner software (Hasting Software, Inc., NY, USA) from RNA sequences from Gene Bank myeloperoxidase (MPO) forward (5′-ACCTACCCCAGTACCGATCC-3′) and reverse (5′-AACTCTCCAGCTGGCAAAAA-3′) primers and P-actin: forward (5′-TCT GGC ACC ACA CCT TCT ACA ATG-3′) and reverse (5′-AGC ACA GCC TGG ATA GCA ACG-3′). All primer sets had a calculated annealing temperature of 60°C. Quantitative RT-PCR was performed in duplicate in a 25 μl reaction volume consisting of 12.5 μl 2x SibriHot Master Mix (BIORON GmbH, Ludwigshafen, Germany), 1 μl of each primer, 5 μl of cDNA and 5.5 μl RNAse-free water using the Applied Biosystem Step One Plus^TM^ RT-PCR system thermal cycling block. The amplification conditions were 2 min at 50°C and 40 cycles of denaturation at 95°C for 15 s and annealing at 60°C/extension at 72°C for 1 min. Data from the real-time assays were calculated using the v17 Sequence Detection Software from PE Biosystems (CA, USA). The relative expression of MPO was calculated using the comparative Ct method. All values were normalized to the P-actin gene [22].

### Statistical analysis

Statistical analysis was induced using a one-way analysis of variance. It was done to compare the control and all other treated groups and was followed by a *post hoc* analysis (Dunnett's test) using the Statistical Package for the Social Sciences, version 17 [23]. The data were presented as the mean ± standard deviation. The difference was considered statistically significant when p < 0.05.

#### Fluorescent microscope examination

Five microns of paraffin-embedded, unstained lung sections were prepared and deparafinized then examined using fluorescent microscopy to ensure homing of labeled-MSC cells in the lung tissues, which had an excitation of 551 nm and an emission of 567 nm.

#### Immunohistochemistry method (CD105 immunostaining)

The immunohistochemistry method used DakoCytomation's Envision system and a polyclonal primary rabbit CD105 antibody (Biorbyt, Cambridge, England). Five-micron thick serial sections were prepared and placed on positively charged slides. Antigen retrieval was performed using programmed a PT-Link containing Envision^™^ FLEX target retrieval solution, High pH (50×), for 20 min. Afterward, the sections were treated with 100 μl of primary CD105 antibody (1:200) except for the negative control slides (100 μl of antibody diluents were applied without any primary antibody) at 4°C overnight. After washing in the Envision^™^ FLEX Wash Buffer (Bioscience, Erembodegem, Belgium), and treated with the Envision^™^ FLEX/HRP Buffer (Bioscience) for 30 min. The sections were stained with Mayer's hematoxylin for 3–5 min [24]. Control staining was performed, and no positive staining was found on the control slides.

#### Histopathological examination

Lung specimens from all groups were fixed, in 10% neutral buffer formalin after intratracheal inflation of the lung with 10% neutral buffer formalin, then dehydrated in alcohol and prepared in paraffin sections. Five-micron sections were prepared and stained using Harris hematoxylin and eosin and Masson's trichrome stain [25] for the histopathological examination.

## Results

### Identification of MSCs in mice

The microscopic examination for flushing and determining the centrifugation BM yield just before the incubation showed patches of aggregated cells. However, after 3 days of incubation, elongated, fusiform and spindle-shaped cells were extensively proliferated and adhered to the wall of the flask. A relatively homogenous cell culture of BM-MSCs with what resembled fibroblast morphology appeared after 7 days of incubation. The cell population reached 70–80% confluence on the 14th day of incubation with a fibroblastoid morphology (Figure 1).

The identification and purification of MSCs by flow cytometric analysis based on cell surface marker expression detected that MSCs were uniformly negative for CD45 and positive for CD29, CD90 and CD105 (Figure 2).

### Fluorescent microscope results

Fluorescence microscopy for the slides prepared from lung sections after deparaffinization with unstained sections and no treatment showed no auto-fluorescence for both gp 1 and gp 2. Red fluorescent spots (MSCs labeled with PKH26 fluorescent dye) were displayed in gp 3, confirming the movement of these cells into the lung tissues on the 1st, 7th and 14th DPI with the most diffuse concentration on the 7th DPI (Figure 3).

### Proinflammatory (TNF- α) & anti-inflammatory (IL10) levels in the plasma & BAL

#### Quantitative ELISA analysis of TNF-α in both (plasma & BAL)

##### TNF-α level in Plasma

As shown in Figure 4, the mean level of plasma TNF-α was significantly increased in gp 2 at 1st and 7th DPI (p < 0.001) when compared with the control group. Similarly, the mean level of plasma TNF-α was significantly increased in gp 3 on the 1st DPI (p < 0.001) and the 7th DPI (p < 0.05) when compared with the control group. In contrast, it was significantly decreased in gp 3 on the 1st and 7th DPI (p < 0.001) when compared with gp 2. Meanwhile, on the 14th DPI, gp 2 (p = 0.650) and gp 3 (p = 0.435) were significantly decreased and approximately reached the parameters of gp 1.

##### TNF-α level in BAL

As shown in Figure 5, the mean level of BAL TNF-α was significantly increased (p < 0.001) in gp 2 on the 1st and 7th DPI when compared with the control group. Moreover, the mean BAL TNF-α level in gp 3 had significantly decreased (p < 0.001) on the 1st and 7th DPI when compared with gp 2, but no significance appeared on the 1st and 7th DPI (p = 0.507 and p = 0.437, respectively) when compared with the control group. In addition, BAL TNF-α was nonsignificantly changed in gp 2 (p = 0.303) and gp 3 (p = 0.832) on the 14th DPI when compared with the control group.

#### Quantitative ELISA analysis of IL-10 in plasma & BAL

##### IL-10 level in plasma

As shown in Figure 6, the mean level of plasma IL-10 was nonsignificantly changed in gp 2 on the 1st, 7th and 14th DPI (p = 0.102, p = 0.775 and p = 0.132, respectively) when compared with the control group. Moreover, the mean level of plasma IL-10 was significantly increased (p < 0.001) in gp 3 on the 1st, 7th and 14th DPI when compared with the control group and gp 2. The mean level of plasma IL-10 in gp 3 on the 14th DPI was significantly increased (p < 0.001) when compared with that of the 1st and 7th DPI in the same group.

##### IL-10 level in BAL

As shown in Figure 7, the mean level of BAL IL-10 was significantly decreased (p < 0.001) in gp 2 on the 1st, 7th and 14th DPI when compared with the control group. In contrast, gp 3's mean level of BAL IL-10 had significantly increased on the 1st, 7th and 14th DPI (p < 0.005, p < 0.001 and p < 0.001, respectively) when compared with gp 2. While the mean level of BAL IL-10 in gp 3 was significantly decreased on the 1st DPI (p < 0.001) when compared with the control group, no significant change was observed on the 7th DPI (p = 0.241) or the 14th DPI (p = 0.147) in gp 3 compared with gp 1.

### Quantitative RT-PCR for myeloperoxidase gene expression in lung tissue

As shown in Figure 8, the mean MPO gene expression in the lung was significantly increased on the 1st, 7th and 14th DPI (p < 0.002, p < 0.001 and p < 0.001, respectively) in gp 2 when compared with the control group. At the same time, the mean *MPO* gene expression in gp 3 was nonsignificantly changed at 1st and 7th DPI (p = 0.240 and p = 0.057, respectively) in comparison with the control group, while a slightly significant increase showed at 14th DPI (p < 0.05) when compared with the control group. Moreover, the mean *MPO* gene expression in gp 3 was significantly decreased in gp 3 on the 1st, 7th and 14th DPI (p < 0.05, p < 0.002 and p < 0.001, respectively, when compared with gp 2. Meanwhile, no significant difference was identified in the mean *MPO* gene expression in gp 3 for the 7th and 14th DPI (p = 0.231 and p = 0.170) when compared with the same group for the 1st DPI.

### Immunohistochemistry results

The microscopic appearance of lung tissues stained by CD105 antibody for BM-MSCs showed no reaction (brown granules) in the pulmonary tissues in the noninfected mice (gp 1) on the 1st, 7th and 14th DPI (without or with application of primary antibody) (Figure 9A–C). The same appearance with no positive reaction for peroxidase activity was detected in the infected pulmonary tissues in LPS-infected mice (gp 2) on the 1st, 7th and 14th DPI, respectively (Figure 9D–F). However, the lungs in gp 3, which was treated with BM-derived MSCs on the 1st DPI, displayed a positive granular brown reaction inside the lumen of perialveolar blood vessels and surrounded the alveolar tissue (Figure 9G). All cases of LPS infected mice treated with BM-derived MSCs on the 7th DPI noticed that MSCs formed into spindle-shaped cells and replaced most of the alveolar epithelial cells and large brown cells (activated macrophages with positive granular brown reaction in their cytoplasm in peribronchiolars and among the septal cells; Figure 9H). The same group on the 14th DPI detected a brownish coloration (macrophages) and a brown color reaction. MSCs covered most of the pulmonary tissues and surrounded the bronchioles inside the peribronchial blood vessels (Figure 9I).

### Pathological results

With respect to the recorded clinical signs, the mortality rate was 0% in all experimental groups. The morbidity rate reached up to 100% in gp 2 (LPS-induced lung injury) in comparison to 0% in gp 1 (control) and variable morbidity rates in gp 3 (MSC-treated), which differed according to the time of treatment (90% on the 1st DPI, 40% on the 7th DPI and <10% on the 14th DPI). Morbidity always included a loss of appetite and a rapid respiratory rate. The microscopic appearance of lung tissues stained with hematoxylin and eosin in noninfected mice (gp1) on the 1st, 7th and 14th DPI showed normal structure of pulmonary tissues (alveoli, bronchi and bronchioles) (Figure 10A–C). The lungs of LPS-infected mice (gp 2) on the 1st DPI showed focal areas of alveolitis with thickening in the septa and proliferation in the epithelial lining of the bronchioles. Edematous lungs found manifested by vesicle densities in type I epithelial cells of the alveolar septa (Figure 10D). The same group displayed severe interstitial pneumonia with emphysema and hemorrhage, besides congestion in all blood vessels on the 7th DPI. Alveolitis was manifested by a thickening of the interalveolar septa with aggregations of inflammatory cells (mainly neutrophils and fibroblasts), besides vasculitis were seen (Figure 10E). However, LPS-infected mice on the 14th DPI displayed inflammatory edema in the lung manifested by vesicle densities in type I epithelial cells of the alveolar septa, with aggregation of inflammatory cells mainly neutrophils among the alveoli (Figure 10F). The common lesions that appeared in these cases were narrowing in the lumen of the bronchioles, congestion in peribronchial blood vessels and inflammatory cells and fibroblasts cells infiltrated among the pulmonary tissue. MSC-treated LPS infected mice (gp 3) on the 1st DPI, displayed signs of lung recovery where the main inflammatory cells, particularly macrophages and a few neutrophils, were scattered among the wall of the bronchioles and the alveolar cells (Figure 10G). MSCs (gp 3) restored alveolar epithelial type I (ATI) cells to their normal structure, on the 7th DPI, where numerous spindle cells light in color filled the alveolar tissues, and the lungs appeared normal in the alveolar and bronchiolar structures in some cases (Figure 10H). In contrast, focal areas of mononuclear cells were detected in the pulmonary tissue, with few emphysematous areas. Multiple aggregations of inflammatory cells, particularly macrophages and neutrophils, are commonly seen in the lung tissues of some animals. Other cases showed a greater degree of progression as indicated by the decline in inflammatory cells at the alveolar septa and nearly normal structure in the lungs tissues. On the 14th DPI, the lungs in gp 3 displayed a proliferation of the peribronchial lymphoid tissues with nearly normal alveolar and bronchiolar structures (Figure 10).

The microscopic appearance of the lung tissues stained by Masson's trichrome in the noninfected mice (gp 1) on the 1st, 7th and 14th DPI indicated that a few collagen fibers were stained blue in color in the peribronchial and perivascular tissues in normal lungs (Figure 11A–C). The injured lungs in gp 2 on the 1st DPI had only a few collagen fibers stained blue in color in the perivascular and peribronchial tissues (Figure 11D). However, the same group on the 7th DPI demonstrated that bundles of collagen fiber had replaced the alveolar cells (Figure 11E). On the 14th DPI, the lungs in all cases in gp 2 showed that collagen fibers stained blue had replaced most of the injured alveolar cells (Figure 11F). The MSC-treated LPS-infected mice (gp 3) displayed few perivascular collagen fibers in the lungs on the 1st DPI (Figure 11G). On the 7th DPI, the injured lungs of the same group displayed normally appearing alveolar tissues with few collagen fibers, perialveolar blood vessels and peribronchial tissues (Figure 10H). The same lesions were displayed in the lungs in gp 3 on the 14th DPI, where few collagen fibers adherent to the bronchial and peribronchial tissue were observed in some mice (Figure 11I).

## Discussion

BM-MSCs played an integral role in the healing of LPS-induced lung injury, which was manifested by edema, alveolitis with deposits of collagen matrix, leukocyte recruitment and bronchiolitis (gp3). Previous studies have shown that some of these roles detected that BM-derived MSCs convert the systemic endotoxin response from a proinflammatory one to an anti-inflammatory milieu by suppressing the generation of proinflammatory mediators without hampering the generation of anti-inflammatory mediators [19]. Our results showed that for the LPS infected group (gp 2) on the 1st and 7th DPI, there are significantly increased plasma and BAL levels of TNF-α as well as MPO gene expression levels. However, in comparison with the control group on the 1st, 7th and 14th DPI, the plasma level of IL-10 showed no significant change in the LPS-treated gp (2), while there was a significant increase in the MSC-treated gp (3). This result confirmed the immuno-modulatory effect of MSCs that alters cytokine secretion and elevates the anti-inflammatory response by increasing the IL-4 secretion and an inhibited the production of TNF-α [26,27]. Additionally, MSCs suppressed endotoxin-induced lung inflammation in acute lung injury by decreasing neutrophilic infiltration and edema.

In addition, MSCs inhibit inflammatory cells and suppress the generation of proinflammatory mediators (such as TNF-α), while maintaining a local BAL level of IL-10, which contributes to lung injury repair [19]. MSCs significantly decreased collagen deposition, which can be attributed to the action of MSCs in blocking the production of TNF-α and IL-1 that mediate fibrotic lung injury [28]. MSCs secrete factors to upregulate the secretion of IL-10 via peripheral blood mononuclear cells, tolerogenic macrophages [29] and tolerogenic DCs [30–32]. BM-derived MSC have the ability to engraft and differentiate into specific and distinct lung cell phenotypes, associated with the suppression of inflammation, a reduction in the total neutrophils counts, a decrease in inflammatory cytokines (TNF-α) and triggering the production of reparative growth factors, to protect the lungs from injury and fibrosis [33,34].

To confirm the action of MSCs inside lung tissue, we looked for the presence of MSCs in lung tissue by using fluorescent detection which clarifies that the PKH26-labeled MSCs in gp 3 were attracted to this tissue after the 1st DPI and remained in the lung until the 14th DPI. These results substantiate the ability of cells to migrate and set up long-term engraftment at the site of injury after the intravenous injection of MSCs [35]. In contrast, PKH26-labeled MSCs were detected in the blood and lungs at 2–6 h after the injection [36]. Furthermore, MSCs homed in on damaged tissues by moving from the bloodstream to inflammatory sites via the utilization of adhesion molecules, such as selectins and integrins, and chemokines and their receptors [37]. Moreover, MSCs express a large range of receptor tyrosine kinase growth factors, such as PDGF or IGF-1, to home in on MSCs [38].

By immunohistochemistry staining using a CD105 antibody (a surface marker for MSCs), our studies tracked the progress of MSCs inside lung tissues to discover other modulatory roles for MSCs in lung tissue healing. The CD105 antibody is used to screen and determine tissue-specific incorporation of donor-derived cells in recipient animals [39]. Our work indicated that spindle-shaped cells (with cytoplasmic brown granules confirming the presence of MSCs) were in the intravascular space and dispersed among septal cells in tissues from MSC-treated mice (gp 3), particularly on the 1st DPI, and in a distributed pattern on the 14th DPI. MSC homing was first localized inside the lumen of the alveolar blood vessels on the 1st DPI and then MSCs migrated and covered all damaged lung tissues on the 7th and 14th DPI. Transplanted MSCs interact with the blood vessel wall during extravasation. MSCs exhibited rolling and firm adhesion onto endothelial cells (ECs), which increased when ECs were prestimulated with TNF-α and binding was via P-selectin *in vivo* [40]. Alveolar capillary ECs play a role in promoting alveolar regeneration [41]. A previous study showed that adult stem cells can undergo both self-renewal and differentiation in multiple lineages, and from these properties, it was suggested that BM-derived stem cells could repair damaged tissues by differentiating intoepithelial cells in disparate sites [34]. *In vitro* differentiation studies have demonstrated the potential of MSCs to differentiate into alveolar and airway epithelial cells [42,43].

Histopathology confirmed the action of MSC in the pulmonary tissue of gp 3 mice in response to LPS-induced lung injury, which caused many changes, including alveolar edema manifested by vesicle densities in type I epithelial cells of the alveolar septa on the 1st, 7th and 14th DPI. At the 1st DPI, focal areas of alveolitis were evident from diffuse neutrophil infiltration and few macrophage cells associated with bundles of collagen fibers (confirmed by Masson's trichrome stain). At the 7th and 14th DPI, there was a progression of interstitial pneumonia. The improvement of MSCs to repair the alveolar and bronchiolar tissues was manifested by reduced inflammation and limited collagen depositions on the 7th and 14th DPI. These positive effects were attributed to the fact that MSCs secrete large quantities of bioactive factors, which suppress the local immune system, inhibit fibrosis and apoptosis, besides enhancing angiogenesis and the differentiation of tissue intrinsic progenitor cells [44]. In addition, one study demonstrated that murine MSCs are home to the lungs in response to injury, adopt an epithelial-like phenotype, and reduce inflammation and collagen deposition in the lung tissue of mice challenged with bleomycin [45]. The reduction in hemorrhage and edema that was observed in MSC-treated gp 3 can be attributed to the preservation of endothelial and epithelial tissue integrity that is mediated by MSCs, which is essential for the maintenance of adequate homeostasis in both the pulmonary and systemic circulations [46]. When MSCs come into contact with injured tissues, they release soluble factors that are capable of modulating cell proliferation [47]. In our work, the endotoxin (LPS) induced changes in lung tissue and the engraftment of MSCs inside lung tissues and adherence to ECs and alveolar tissues is attributed to the MSCs’ ability to help in the initiation of alveolar epithelial type II (ATII) cell activation. ATII cells have been considered to be similar to stem cells in adult lungs, and they behave as progenitor cells by proliferating and differentiating into type I cells following injury, during alveolar homeostasis and during repair [48–51]. Developmental studies have confirmed that the progenitor cell properties of ATII cells are controlled by the cell matrix and cell–cell interactions related to their particular environments before or after injury [52–55]. It is not clear if these putative regenerative ATII cells, which appear postinjury, are derived from the expansion of existing stem cell pools located in an undefined niche or are derived from quiescent, terminally differentiated ATII cells. Therefore, it would be interesting to identify which signals or factors induce the formation of progenitor-like ATII cell subgroups following injury [56]. BM-derived cells are capable of forming lung alveolar epithelium, and it was demonstrated that cultured BM cells could act as type I pneumocyte precursors [57].

In contrast, our findings suggest that ATI cells are the most affected by LPS (endotoxin) leading to changes in the extracellular matrix. A solution to LPS-induced lung tissue injury was evident by lung tissue improvement in the majority of mice treated with MSCs derived from BM (gp 3). We conclude that MSCs may have progenitor cell properties such as ATII cells or that they may be able to serve as ATII that can convert to ATI cells in order to remodel lung tissue.

## Conclusion

MSCs could regulate inflammatory cytokines through the accumulation of macrophages that stimulated the anti-inflammatory activity of IL-10 and suppressed TNF-α by a reduction of neutrophils in lung inflammation. In addition, MSCs probably act as progenitors for remodeling alveolar tissues and prevent the fibrosis, as well as, ATII cells that can convert to ATI cells.

## Future perspective

Our work reported on the remodeling effect of MSCs on acute lung injury. In the future, this study may allow us to look for the effect of MSCs on treat chronic lungs injury and will progress to how it can lysis the fibrosis in lungs by using MSCs in gene therapy.

**Executive summary**

Lipopolysaccharide (LPS) also plays an important role in the pathogenesis of acute lung injury, resulting in significant morbidity and mortality in both humans and animals.

**Regeneration of lung tissue by bone marrow-derived mesenchymals**

We evaluated the modulatory effect of bone marrow-derived mesenchymals (BM-MSCs) on inflammatory cytokines, including TNF-α and IL 10, and their differentiation into alveolar type I (ATI) cells through their direct contact with injured murine lung tissue. MSCs in culture were characterized by CD29, CD90 and CD105 surface markers of mouse MSCs using flow cytometry.

**Experimental design on mice**

Sixty-three mice were divided into three groups (n = 31). The first group was the control group. The second group was inoculated with one 0.1-ml intraperitoneal dose of 0.025 mg LPS/mouse. The third group was treated with intravenous injection with one dose of labeling (BM-MSCs) at (7.5 × 10^5^ cell/mouse), 4 h post-inoculation with (0.1 ml LPS/mouse). All mice were euthanized on the 1st, 7th and 14th days post injection. Blood, bronchoalveolar lavages (BALs) samples and lung tissues were collected to measure levels of IL-10, TNF-α and myeloperoxidase with real-time PCR, histopathological and immunohistochemistry analysis, respectively.

**Alternative effects of cytokines in the presence of BM-MSCs**

The levels of proinflammatory cytokines (TNF-α) were significantly decreased in BAL and plasma, whereas the levels of anti-inflammatory cytokines (IL-10) were significantly higher in BAL and plasma in MSC-treated LPS-infected group (gp 3). This finding confirmed the immuno-modulatory effect of MSCs on LPS-induced lung injury.

**Histopathological & immunohistochemistry results from of the LPS-induced lung injury & MSCs treated lung injury**

The lungs in the LPS-infected group (gp 2) exhibited alveolitis, interstitial edema and interstitial pneumonitis, in addition to vesicle densities in ATI cells from all sacrificed animals on the 1st, 7th and 14th DPI. The lungs in the MSC-treated LPS-infected lung, displayed a decrease in pulmonary edema and a decrease in collagen fibers with a renewal in ATI cells leading to fast recovery.

**Conclusion**

MSCs showed an ability to both modulate inflammatory cytokines (TNF-α and IL-10) and to differentiate into cells, which lead to the prevention of lung fibrosis in mice.
